# Predicting clinical outcomes in COVID-19 using radiomics on chest radiographs

**DOI:** 10.1259/bjr.20210221

**Published:** 2022-07-08

**Authors:** Bino Abel Varghese, Heeseop Shin, Bhushan Desai, Ali Gholamrezanezhad, Xiaomeng Lei, Melissa Perkins, Assad Oberai, Neha Nanda, Steven Cen, Vinay Duddalwar

**Affiliations:** 1 Keck School of Medicine, University of Southern California, CA, USA; 2 Viterbi School of Engineering, University of Southern California, Los Angeles, CA, USA

## Abstract

**Objectives:**

For optimal utilization of healthcare resources, there is a critical need for early identification of COVID-19 patients at risk of poor prognosis as defined by the need for intensive unit care and mechanical ventilation. We tested the feasibility of chest X-ray (CXR)-based radiomics metrics to develop machine-learning algorithms for predicting patients with poor outcomes.

**Methods:**

In this Institutional Review Board (IRB) approved, Health Insurance Portability and Accountability Act (HIPAA) compliant, retrospective study, we evaluated CXRs performed around the time of admission from 167 COVID-19 patients. Of the 167 patients, 68 (40.72%) required intensive care during their stay, 45 (26.95%) required intubation, and 25 (14.97%) died. Lung opacities were manually segmented using ITK-SNAP (open-source software). CaPTk (open-source software) was used to perform 2D radiomics analysis.

**Results:**

Of all the algorithms considered, the AdaBoost classifier performed the best with AUC = 0.72 to predict the need for intubation, AUC = 0.71 to predict death, and AUC = 0.61 to predict the need for admission to the intensive care unit (ICU). AdaBoost had similar performance with ElasticNet in predicting the need for admission to ICU. Analysis of the key radiomic metrics that drive model prediction and performance showed the importance of first-order texture metrics compared to other radiomics panel metrics. Using a Venn-diagram analysis, two first-order texture metrics and one second-order texture metric that consistently played an important role in driving model performance in all three outcome predictions were identified.

**Conclusions:**

Considering the quantitative nature and reliability of radiomic metrics, they can be used prospectively as prognostic markers to individualize treatment plans for COVID-19 patients and also assist with healthcare resource management.

**Advances in knowledge:**

We report on the performance of CXR-based imaging metrics extracted from RT-PCR positive COVID-19 patients at admission to develop machine-learning algorithms for predicting the need for ICU, the need for intubation, and mortality, respectively.

## Introduction

As of January 2021, the overall number of global COVID-19 cases is more than 100 million, while the deaths have increased to over 2 million.^1^ Currently, the US accounts for the highest number of severe acute respiratory syndrome coronavirus 2 (SARS-CoV-2) infections and SARS-CoV-2-related fatalities at more than 25 million and 450,000, respectively. Since the beginning of the COVID-19 pandemic, reverse-transcription PCR (RT-PCR) tests have been widely used and remain the primary tool for COVID-19 diagnosis.^2–4^ While most RT-PCR confirmed COVID-19 positive patients have mild and manageable flu-like symptoms, up to 11% develop severe disease requiring hospitalization.^3,5,6^ Of these, 25% need ICU admission.

With a surge in cases, guidelines from oversight committees planning intensive care unit (ICU) resource management strategies expect one in five hospitalized adult COVID-19 positive patients to require ICU admission.^7^ Of these, 70% of ICU patients will require some ventilatory support, with  > 50% of ICU patients requiring invasive ventilatory support. Given the overwhelming demand for critical and limited resources such as ICU beds, mechanical ventilators, etc., it is prudent to identify patient-level characteristics at the time of admission to predict the need for these resources in COVID-19 patients. This will help to design surge capacity resource planning.

Recently, Artificial Intelligence (AI) systems for the diagnosis of COVID-19 on Chest X-ray (CXR) or chest CT have been tested with variable accuracy.^8–12^ Thus, we aimed to validate the utility of quantitative CXR radiomics metrics in patients with RT-PCR confirmed COVID-19 infection in indiscriminately differentiating between patients with poor *vs* good outcomes. Here, poor outcomes are defined as the need for ICU admission, the need for intubation, and a higher risk of fatal outcomes in hospitalized patients. Based on current literature, we believe that CXRs contain predictive information of clinical outcomes in patients who develop severe symptoms of COVID-19.^13,14^ Therefore, we hypothesize that using CXR-based radiomics with machine-learning, we can predict the need for ICU, intubation, and death among hospitalized patients.

Our study is unique as it focuses on quantitative extraction of imaging metrics from clinically acquired CXR, which is part of the standard of care (SOC) management of COVID-19 patients in the USA to predict clinical outcomes. We consider data from Los Angeles County representing a multiethnic patient population in training our model. This is relevant since the outcome for COVID-19 is known to be dependent on demographics.^15,16^

## Methods and materials

This project’s data were obtained from an IRB-approved COVID-19 imaging repository set up at our institution. This COVID-19 repository collects and stores imaging and associated clinical data from all RT-PCR positive COVID-19 patients in a University of Southern California hosted REDCap database. A waiver for informed consent was obtained. Subject’s privacy and confidentiality were protected according to applicable HIPAA and Institutional IRB policies and procedures.

## Patient cohort

Our study identified 167 patients (mean age 55.4 years; range 19–93 years) between March and May 2020, with radiographic findings on CXRs available within ± 2 days from RT-PCR date. Our study cohort was obtained from three hospitals (one public teaching, one private teaching and one private community). Of the 167 patients, 107 were males, and 59 were females, and one other. Of the 167 patients, 68 (40.72%) required intensive care during their stay, 45 (26.95%) required intubation, and 25 (14.97%) died. An experienced research coordinator (MP) conducted chart reviews on all patients enrolled in the COVID-19 repository. Our inclusion criteria restricted enrollment to only patients with a confirmed diagnosis of COVID-19 by RT-PCR and a radiologic finding on the CXR. Details of patient demographic information has been provided in Table 1.

**Table 1.** Description of patient cohort. Age reported as mean, median (interquartile range). All other variables reported as sample size (percentage)

Variables	Sample size
**No. of patients (n)**	167
**Age**	55 ± 17, 55 (43 to 68)
**Sex**	
Male	107 (64.07%)
Female	59 (35.33%)
Other	1 (0.6%)
**Ethnicity**	
Hispanic Latino	111 (66.47%)
Non-Latino	40 (23.95%)
Unknown	16 (9.58%)
**Mortality**	
Survived	142 (85.03%)
Deceased	25 (14.97%)
**ICU Admission**	
No ICU	99 (59.28%)
ICU	68 (40.72%)
**Intubation**	
No intubation	122 (73.05%)
Intubation	45 (26.95%)

## CXR acquisitions

Our analysis included one CXR per patient for a total of 167 CXRs. All these images were community-acquired as part of creating a COVID-19 repository. Not all patients had a computed tomography (CT) to correlate with the CXR. The repository contains demographic, imaging, clinical and laboratory data for all COVID-19 positive patients seen at three different sites, namely, the Keck Medical Center of USC, Verdugo Hills Hospital, and Los Angeles County + USC Medical Center. Of the collected data, we used the CXR images closest to the date of RT-PCR-confirmation. We also used the outcome data, viz, the need for ICU admission, intubation, and death. All studies were uploaded to and accessible from our institutions’ picture archival and communications systems (PACS) as part of clinical SOC at the time of enrollment.

## Radiomics analysis

**Figure 1.**
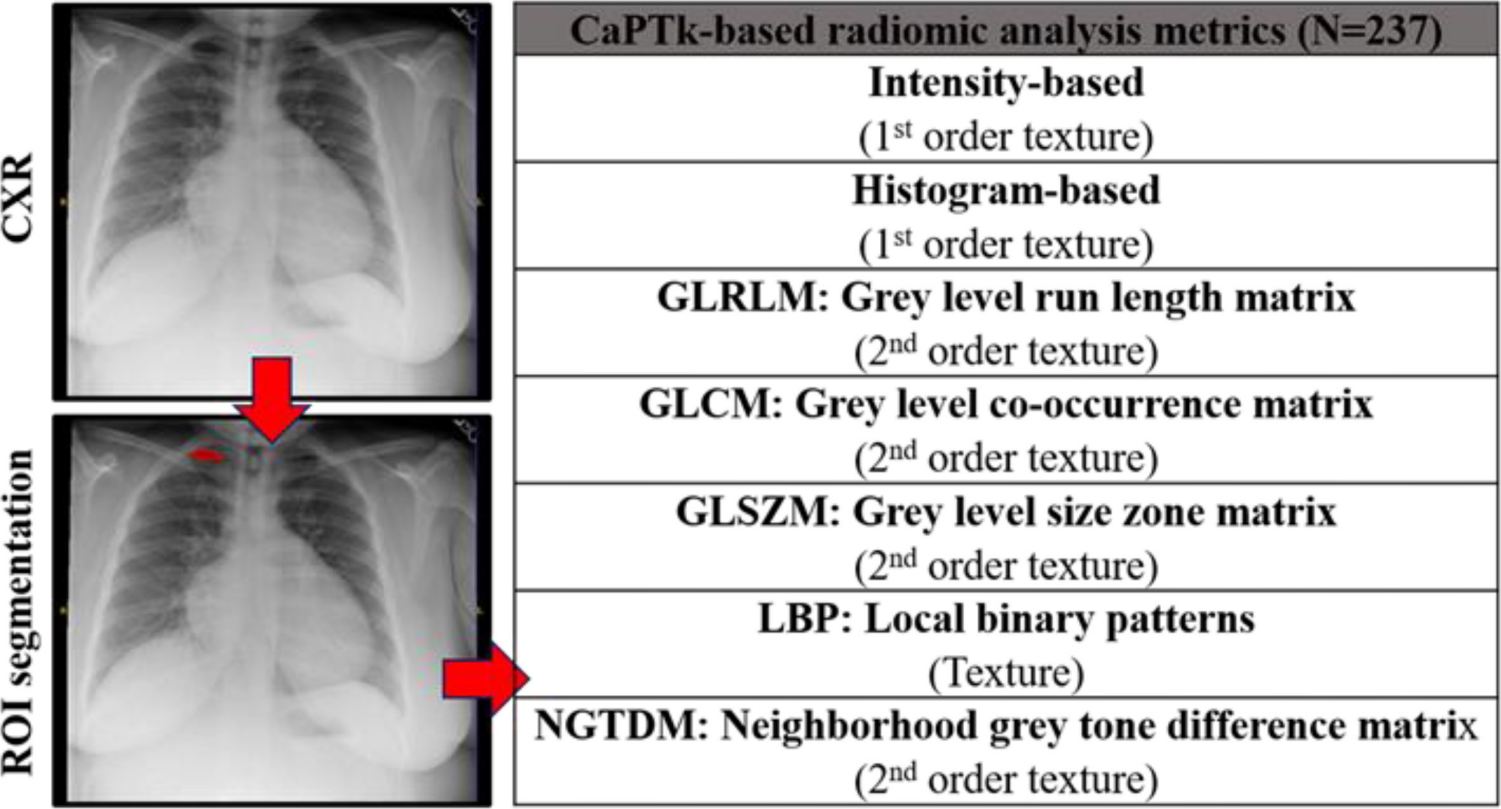
A typical radiomics workflow showing its four main Stages 1. Image acquisition 2. Segmentation and/or ROI marking (highlighted in red) 3. Feature extraction and finally 4. Statistical analysis. The two green axes divide the image plane into four quadrants. We use 237 radiomic metrics across seven different texture families for this study.

For radiomics analysis, the single largest lung abnormality was identified on the CXR per patient. The opacities on the CXRs were manually segmented by HS (>5 years of experience in diagnostic radiology) using a two-dimensional region of interest (ROI) that was placed contouring the identified lung abnormality using ITK-SNAP (open-source software; http://www.itk.snap.org). If no lung abnormality was apparent, then segmentation was not performed. All the 167 patients within our cohort had lung opacities. Care was taken to avoid artifacts, such as ribs, wires, etc. The resultant ROIs from the ITK-SNAP segmentations were saved for transfer, processing, and radiomic analysis (Figure 1). Cancer Imaging Phenomics Toolkit (CaPTk),^17–19^ an open-source software platform (https://www.med.upenn.edu/cbica/captk/), was used for feature extraction of ROIs obtained following ITK-SNAP extraction of tumor lesions. These segmented ROIs were then transferred to CaPTk for radiomics analysis (Table 2). Only 2D radiomics analysis of texture was performed. The radiomics metrics quantify the complex relationship between pixels/voxels making up the region of interest (texture) using sophisticated data characterization algorithms. Some of the key metrics featured within the CaPTk radiomics platform include first-order statistical metrics of texture, such as intensity, histogram; second-order statistical metrics of texture, such as Grey level co-occurrence matrix (GLCM), Grey level size zone matrix (GLSZM), Grey level run length matrix (GLRLM), and higher order statistical metrics of texture such as Local binary patterns (LBP). First-order statistical metrics quantify only the ’signal’s intensity within a region of interest. Second-order statistical metrics consider the intensity and spatial orientation, location within a region of interest. Higher order metrics transform the image to provide additional information regarding frequency, assessment at multiple levels (local versus global assessment). All results were exported in comma-separated value (.csv) format for radiomics analysis.

**Table 2.** Radiomic Features Evaluated on Diagnostic CXR in 167 COVID-19 positive patients. Radiomic features extracted by CaPTk (Cancer Imaging Phenomics Toolkit). CaPTk provides quantitative imaging analytics for precision diagnostics and predictive modeling of clinical outcomes. All features of CapTk are in conformance with the Image Biomarker Standardization Initiative (IBSI), unless otherwise indicated within the documentation of CaPTk.^19^ Additional details on the definition equation and implementation of these metrics in CaPTk can be found here: https://cbicagithubio/CaPTk/tr_FeatureExtractionhtml#tr_fe_defaults. From all the radiomic metrics available on CaPTk, only the 2D radiomic metrics of texture were calculated

Family	Metric	What it measures
**Intensity-based** (**Texture**)	Minimum Intensity	These features quantify the distribution of the grey-levels (histogram) making up the region of interest. It provides distribution of the histogram. These are **first-order statistical** metrics of radiological texture, as it accounts for only the grey-level intensity in the image, not its spatial orientation of the image.
	Maximum Intensity	
	Mean Intensity	
	Standard Deviation	
	Variance	
	Skewness	
	Kurtosis	
**Histogram-based** (**Texture**)	Bin frequency & probability	These features quantify the distribution of the grey-levels (histogram) making up the region of interest. These are **first-order statistical** metric of radiological texture.
	Intensity values (fifth quantiles)	
	Intensity values (95^th^ quantiles)	
	Bin-level statistics	
**GLRLM: Grey level run length matrix** (**Texture**)	SRE: Short Run Emphasis	These metrics quantify the relationships between image pixels/voxels. In GLRLM analysis, texture is quantified as a pattern of grey-level intensity pixel in a fixed direction from a reference pixel. Run-length is the number of adjacent pixels with the same gray-level intensity in each direction. These are **second-order statistical** metrics of radiological texture as it accounts for both the grey-level intensity and its spatial orientation of the image.
	LRE: Long Run Emphasis	
	GLN: Grey Level Non-uniformity	
	RLN: Run Length Non-uniformity	
	LGRE: Low Grey Level Run Emphasis	
	HGRE: High Grey Level Run Emphasis	
	SRLGE: Short Run Low Grey Level Emphasis	
	SRHGE: Short Run High Grey Level Emphasis	
	LRLGE: Long Run Low Grey Level Emphasis	
	LRHGE: Long Run High Grey Level Emphasis	
**GLCM: Grey level co-occurrence matrix** (**Texture**)	Energy	These metrics quantify the relationships between image pixels/voxels. In GLCM analysis, texture is quantified as a tabulation of how often a combination of grey-level values in an image occur next to each other at a given distance in each direction. These are **second-order statistical** metrics of radiological texture.
	Contrast	
	Entropy	
	Homogeneity	
	Correlation	
	Variance	
	SumAverage	
	Variance	
	Autocorrelation	
**GLSZM: Grey level size zone matrix** (**Texture**)	SZE: Small Zone Emphasis	These metrics quantify the relationships between image pixels/voxels. In GLSZM analysis, texture is quantified as a tabulation of how often a combination of grey-level values in an image occur next to each other at a given distance. Contrary to GLCM and GLRLM, GLSZM is direction independent. These are **second-order statistical** metrics of radiological texture.
	LZE: Large Zone Emphasis	
	GLN: Gray Level Non-Uniformity	
	ZSN: Zone-Size Non-Uniformity	
	LGZE: Low Gray Level Zone Emphasis	
	HGZE: High Gray Level Zone Emphasis	
	SZLGE: Small Zone Low Gray Level Emphasis	
	SZHGE: Small Area High Gray Level Emphasis	
	LZLGE: Large Zone Low Gray Level Emphasis	
	LZHGE: Large Zone High Gray Level Emphasis	
	GLV: Gray Level Variance	
	ZV: Zone Variance	
**LBP: Local binary patterns** (**Texture**)	Select first-order and second order texture metrics such as mean, median, standard deviation etc.	These metrics are computed using sampling points on a circle of a given radius and using mapping table. These are **higher-order statistical** metrics of radiological texture.
**NGTDM: Neighborhood grey tone difference matrix** (**Texture**)	Coarseness	These metrics quantify the difference between a gray-level intensity and the average gray-level intensity of its neighborhood within a given distance. These are **second-order statistical** metric of radiological texture.
	Busyness	
	Contrast	
	Complexity	
	Strength	

## Statistical analysis

For demographic information, we have used Chi-square test (for categorical data) and ANOVA or Kruskal–Wallis test (for continuous data) to compare across the patient demographics across the three study sites. Univariate independent t-test or Wilcox on rank sum test depending on data normality, along with mean, standard division, and interquartile range displayed in box plot were used as the descriptive analyses for the association of radiomic features with clinical outcomes. Benjamini-Hochberg (BH) procedure was used to control multiple comparison error for univariate analyses. Percent features with unadjusted and BH procedure adjusted *p* < 0.05 by each radiomics family was calculated as the assessment of overall signal strength from each radiomic family, in comparing to variable of importance generated by machine learning. Three machine learning (ML) algorithms were used to evaluate the ability of radiomic features to predict biomarkers: Random Forest (RF), Real AdaBoost,^20^ and ElasticNet. RF and AdaBoost are considered non-parametric approaches while ElasticNet is considered parametric. For all three classifiers, 10-fold cross-validation was used to evaluate model performance. The full dataset was equally divided into tenfolds. We re-iterated the learning process ten times and applied the classifier to each testing sample. Thus, each study sample served as an independent testing case once. Receiver operating characteristic (ROC) curve was constructed using the predicted probability from 10 testing datasets combined. The area under the curve (AUC) with 95% confidence interval was used to assess prediction accuracy. We applied a fivefold cross-validation (‘out-of-bag’ cross-validation) process within each iteration to determine the final prediction model before scoring through the 10% independent testing sample. The 10% of independent testing data were excluded from the learning phase to avoid information leaking. For Random Forest, we have used 800 trees with a maximal depth of 50, leaf size of 16, and variable to try was the square root of variable number. For Real Adaboost, since it is more efficient, only 25 trees were built with a depth of 3 as recommended by^21^.^21^ For Random Forest and Adaboost, the Gini impurity index was used as the loss function. Loh method^22^ was used for variable selection. This method selected the variable that has the smallest *p*-value of a chi-square test of association in a contingency table; interval variable was truncated by dynamically calculated proportion of standard deviation from mean. Predicted residual sum of squares (CVPRESS) was used for ElasticNet to select candidate predictors and the final model. For imbalanced outcome, prior correction as described by King et.al.^23^ was used. Variable-of-importance (VOI) from Random Forest and Adaboost was selected and ranked using Out-of-bag Gini index (OOBGini), while ElasticNet was the remaining variables in the final model. For Random Forest and Adaboost, the cut-off for top“VOI was determined by the “cliff” of OOBGini, *that is,* a sudden large change from the previous ranking position. The VOI selection procedure was repeated ten times, and the final ranking was based on the number of counts as top VOI during the 10-fold cross-validation. SAS Enterprise Miner 15.1: High-Performance procedures were used for machine learning. SAS9.4 was used for all other statistical analysis.

## Results

In this study, we extracted the texture (spatial distribution of grey levels) content present within the segmented lung opacities using radiomics. There were 237 features extracted for the data analysis. The details of our patient cohort are shown in Table 1.

**Figure 2.**
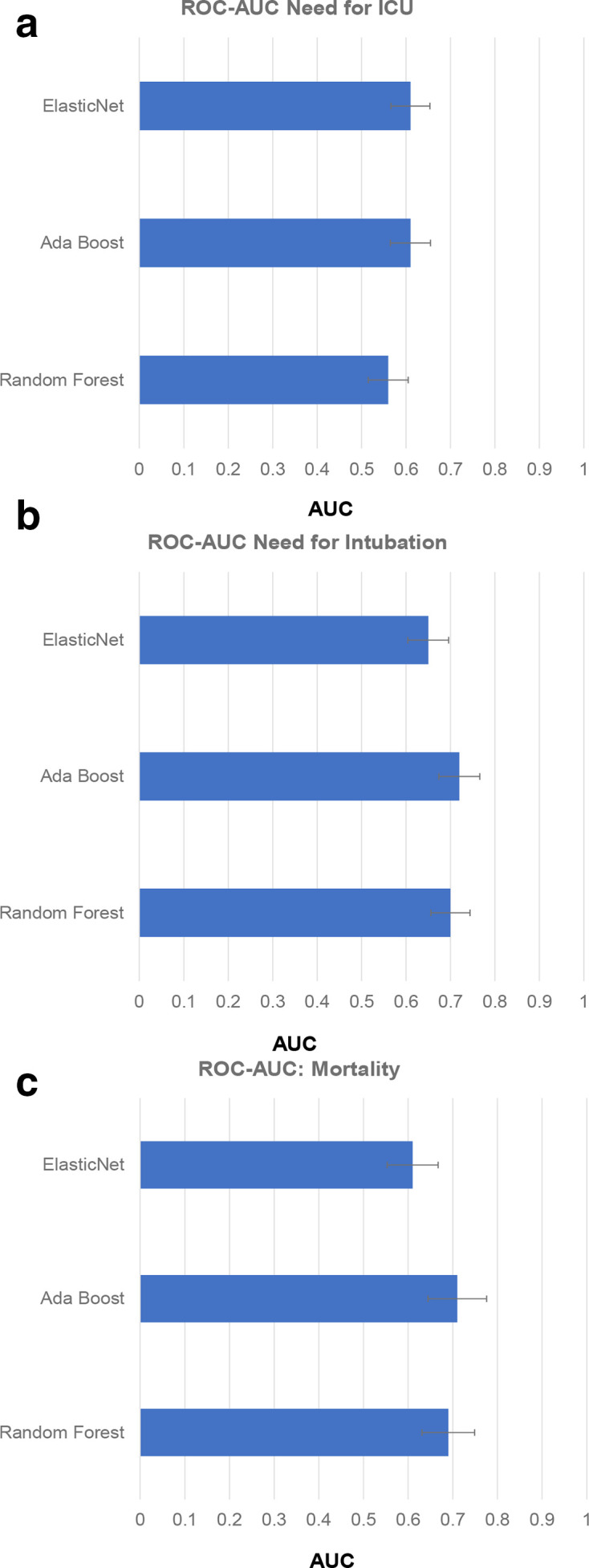
Area under the curve (AUC) plotted along the x-axis for the three classifiers (*i.e.,* Ada Boost, Elastic Net and Random Forest) considered in the study for predicting the need for ICU (**A**), need for intubation (**B**) and mortality (**C**). Of the three classifiers, Ada Boost shows the best performance for predicting the need for intubation and mortality with an AUC of 0.72 and 0.71, respectively. It has similar performance with ElasticNet in predicting ICU with an AUC of 0.61.

In Figure 2, we have plotted the AUC values for predicting the need for ICU, the need for intubation, and death for the three machine learning algorithms considered in this study. This figure illustrates that the Adaboost classifier performs the best in predicting death and intubation. It has similar performance with ElasticNet in predicting the need for ICU.

The associated sensitivity, specificity, positive-predictive value, and negative-predictive value for the three machine learning models across the three outcomes have been reported (Supplementary Material 1).

Supplementary Material 1.

Of the three clinical outcomes, Adaboost-based radiomics signature showed reasonable discrimination (AUC >0.72) for predicting the need for intubation among patients admitted in the ICU. The Adaboost-based radiomic signature reported an AUC of 0.71 and 0.61, respectively, in predicting death and the need for ICU among hospitalized. Our univariate analyses supported these data. 14.7%, 15.2%, and 20.6% of all radiomic metrics reached significance at the *p* ≤ 0.05 level in differing between two groups across the three clinical outcomes, respectively.

Across the seven texture families analyzed, GLRLM yielded the greatest percentage of signatures within a given family to reach significance at the *p* ≤ 0.05 level at 69.38% when assessing the differences in radiomic metrics between hospitalized patients with a higher risk of death compared to others. Similarly, first-order texture metrics such as histogram analysis and intensity yielded the greatest percentage of signatures within a given family to reach significance at the *p* ≤ 0.05 level at 88.9% when assessing the differences in radiomic metrics between ICU patients in need for intubation *vs* their controls. Similar results were also observed when assessing the differences in radiomic metrics between hospitalized patients in need of ICU *vs* their controls.

**Figure 3.**
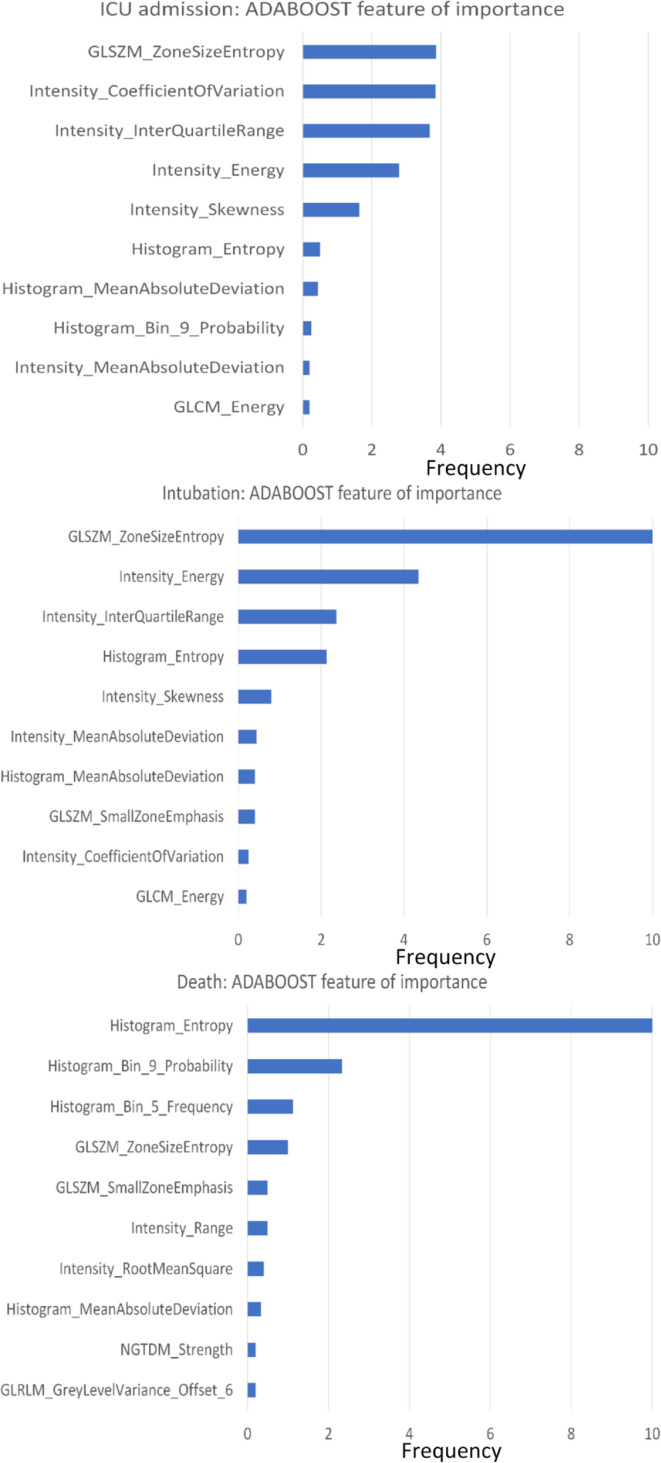
Variable (radiomic metric) of importance is plotted along the y-axis for the Adaboost model across the three outcome predictions *i.e.,* need for ICU, need of intubation and death, respectively based on ranking of radiomic metrics within a rigorous LOO cross-validation procedure. ‘Frequency’ defined as the number of times each variable made to the top 10 variable of importance list during 10-fold cross-validation is plotted along the x-axis.

We used the Out-of-bag Gini index to rank the variables of importance. Of the radiomic metrics, the top 10 that met the criteria for variables of importance for the Adaboost model were from the first-order texture family, such as histogram and intensity, followed by GLSZM and GLCM. This observation was consistent across the three ’outcomes’ prediction models. The distribution of these metrics for Adaboost model is summarized in Figure 3.

**Figure 4.**
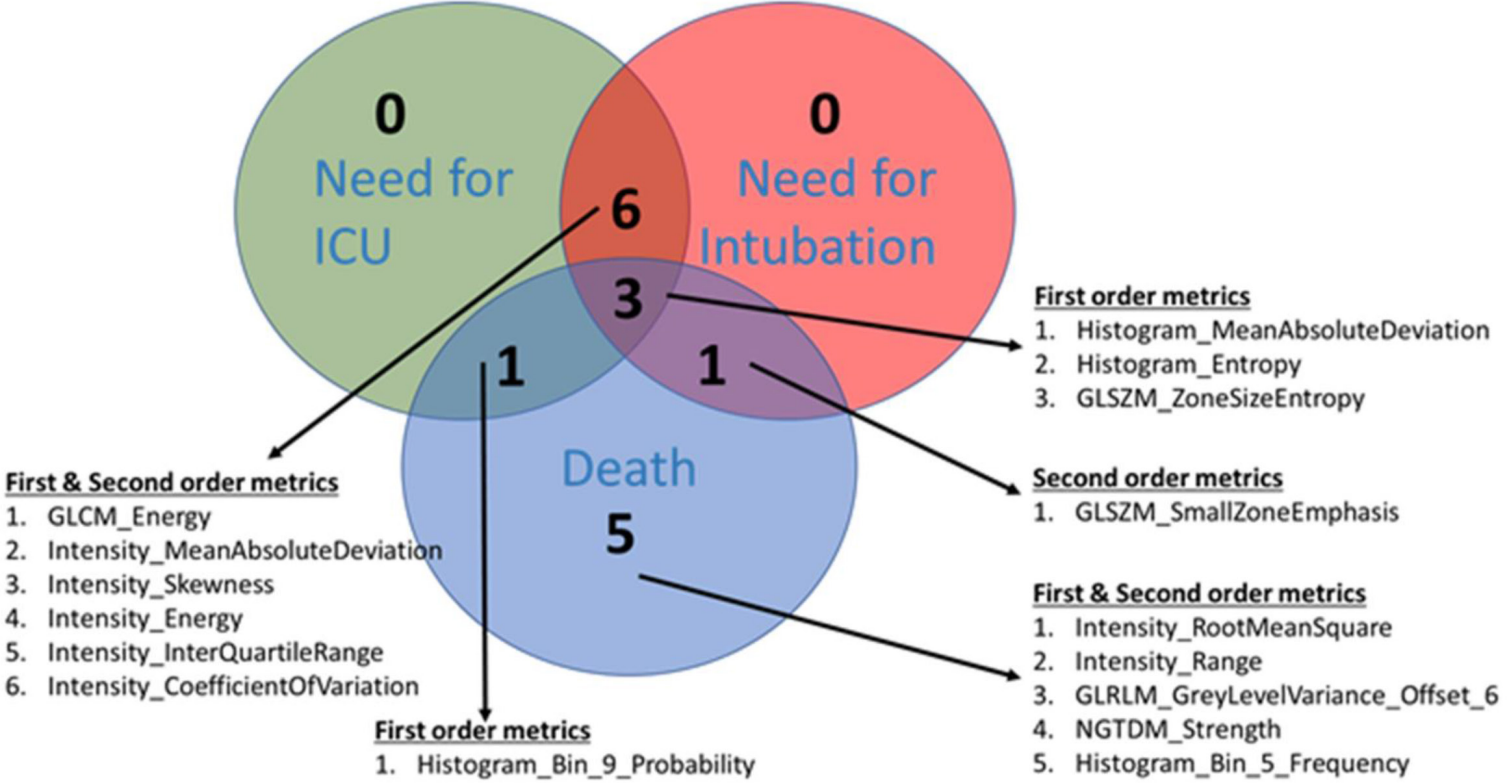
Venn diagram showing the overlap in radiomic metrics between the three prediction models. Of the 3-common overlapping radiomic features across the three prediction models, two belong to the first-order texture metrics: Histogram analysis. The first one, MeanAbsoluteDeviation measures the average distance between each data value and the mean. The metric provides a quantification of the “spread” of the values in a data set. The other histogram metric, entropy help quantifies the information contained within the dataset. Lastly the GLSZM metric: ZoneSizeEntropy evaluates entropy (or uniformity) in the distribution of groups of connected voxels with the same discretized intensity.

A Venn-diagram approach was used to access the overlap of radiomic metrics that made it to the variable of importance criteria for predicting the three outcomes. The results have been summarized in Figure 4. Many overlapping radiomic metrics were identified between the three prediction models. Greater overlap was found between the radiomics metrics driving the prediction model for the need for ICU and need for intubation, compared to other scenarios. At least three different radiomics metrics that met the top 10 variables of importance criteria were common in the three outcomes' prediction models.

## Discussion

Techniques to predict the need for key clinical resources, such as the need for ICU, need for intubation, and high risk of death, are crucial for surge and mass casualty planning, particularly during the COVID-19 pandemic.^24^ Currently, qualitative imaging, clinical, demographic, and blood panel-based metrics are being employed, yet their accuracy varies. Accurate early-on quantitative indicators based on CXR, which is currently part of the standard imaging workup in COVID-19 patients, has the potential to achieve this task. This study utilized a CXR-based radiomics analysis applied to multicenter data to construct such a decision classifier. We used three machine-learning approaches, namely Adaboost, Elastic net, and RF, which yielded reasonable to good discriminative power in predicting clinical outcomes, the need for ICU, the need for intubation, and death. Of the three machine-learning approaches, the best performance was observed using Adaboost. We report AUCs of 0.72 95% CI (0.63 0.81), 0.71 95% CI (0.58 0.84) and 0.61 95% CI (0.49 0.67) in predicting the need for intubation, death and the need for ICU, respectively in 167 RT-PCR confirmed COVID-19 positive patients. In addition to creating prediction models, we also identified radiomic metrics that drive the predication models performance across the three outcomes and investigated the overlap in these metrics. Our results show greater overlap between the radiomics metrics driving the prediction model for the need for ICU and the need for intubation compared to other scenarios. Post-validation in other studies, the results presented in this study demonstrate that data acquired at or around the time of admission of a COVID-19 patient to a care facility may aid in providing an objective and accurate assessment of clinical outcomes, particularly the need for intubation in ICU patients. The presented work is foundational, and we subsequently plan to include lab and clinical metrics for these patients, which may help improve the predictive performance of these models. Central to the hypothesis of radiomics applicability in CXR of COVID-19 patients is its ability to assess lung lesions objectively, such as opacities, and its heterogeneity dynamically in longitudinal studies. The distinctive advantage of CXR radiomics is the comparatively low turn-around time for obtaining results. Despite potential applications of CXR radiomics in COVID-19 assessment, radiomics research has generally been hindered by the search for metrics that are both robust (*i.e.,* stable across multiple image settings) and reproducible (*i.e.,* stable across multiple different scanners).^25,26^ While single-scanner, single-center databases, in theory, provide cleaner analyses, these conditions are not replicable in the real-world environment. For our study, we have included data from three sites with large difference in age and ethnicity distribution (Supplementary Material 1) and used a cross-validation method to assess the model robustness. A 10-fold cross-validation will have had reshuffled the data 10 times and mixed the data from different institutes in both learning and testing sample. This procedure has a similar function as “intend-to-treat” *that is,* to washout the artefact if the prediction model only works for data from one institute/scanner but not others.

CT and CXR have been traditionally used to diagnose and manage viral pneumonia. Since atypical pneumonia is the main presentation of SARS-CoV-2 infection, much has been published using AI-augmented imaging techniques to assess COVID-19; however, these have been mostly limited to CT and mostly using visual assessment and/or semi-quantitative methods. In addition, pathological studies on biopsied lung tissue from COVID-19 patients suggest thrombosis with microangiopathy and vascular angiogenesis to be strong discriminators of the disease.^27^ Therefore, radiomic analysis, particularly texture analysis, which has been shown to capture these phenotypes, could provide more information about COVID-19 as well.^28,29^

Using data from 315 patients, Homayounieh et al reported promising use of chest CT radiomics to differentiate in outpatient *vs* inpatient with an AUC of 0.84 (*p* < 0.005), which was an improvement from “the ’radiologists’ interpretations of disease extent and opacity type, which had an AUC of 0.69 (*p* < 0.0001).^30^ The group also reported an improvement in the performance of radiomics based prediction model as opposed to radiologist predictions in need for ICU admission (AUC:0.75 *vs* 0.68) and death (AUC:0.81 *vs* 0.68) (*p* < 0.002). Yue et al reported CT radiomics models based on a signature of 6 second-order radiomic metrics that discriminated short- and long-term hospital stay in 52 COVID-19 patients, with areas under the curves of 0.97 (95%CI 0.83–1.0) and 0.92 (95%CI 0.67–1.0) by logistic regression and random forest, respectively.^31^ While the performance of CT is better than CXR in assessing COVID-19 infections,^32^ CXR is easier to perform in ICU patients and recommended by various societies in the USA. However, most of the radiomics studies using CXR data revolved around detecting COVID-19 from other viral infections.

Based on our Venn-diagram approach overlapping radiomic metrics were identified between the three prediction models. Greater overlap was found between the radiomics metrics driving the prediction model for the need for ICU and need for intubation, compared to other scenarios. Six different radiomic metrics, five from the first-order family, namely intensity, and one from the second order family, namely GLCM, were found overlapping between the radiomics metrics driving the prediction model for the need for ICU and need for intubation. Interestingly, all the overlapping metrics belong to texture families assessing spatial heterogeneity in grey-levels within the segmented ROI. The overlapping metrics between the need for ICU and need from intubation show the close association of changes in textures as a patient in the ICU eventually needs intubation. These can be detected from CXR scans taken at admission.

While statistical harmonization methods such as ComBat have been used to harmonize scans from different scanners and/or acquisition settings.^33^ A recent study showed when scans been harmonized, it may not preserve the original effect size. In some scenarios, it may introduce “unwanted effects”, which may exaggerate the prediction accuracy.^34^ When the learning and independent testing data been “harmonized” together, the independent data are no longer truly “independent” and may be the reason for the improved accuracy when using ComBat. To the best of our knowledge, no study has examined the change of false discovery when using ComBat. While harmonization of radiomic metrics aid in the reliable assessment of radiomics using multicenter data, not all radiomic features require harmonization. Based on previously published radiomics reliability studies using custom-built CT-radiomics phantoms, we identified radiomic features that were robust without the necessity of harmonization.^25,26^ During cross-validation, the robust features were much more likely to be retained as important predictors. The features with large difference between institutes and scanners would drop out since the unstable signals mostly cancel each other. We have conducted sensitivity analysis using robust feature only in this study (Supplementary Material 1). The prediction accuracy using only the robust features remained similar to the full model using all features. Therefore, while conservative our results are robust.

Our study is not without limitations. All CXR images were segmented manually, which might not be feasible in analyzing very large datasets. The segmented ROIs were the largest most representative captures of the ground glass opacities that were visually observed on the CXR. The exact shape of the ROI was not critical and hence not evaluated in the analysis. Considering CXRs were obtained from multiple sites including portable units, an absolute size assessment of the ROIs was not practical. In subsequent studies, we intend to perform a semi-quantitative assessment of ROI size. However, despite these limitations, this study can lay the foundation for future research in this field. Our results can be used to develop more accurate tools for identifying early predictors of poor outcomes such as the need for intubation, ICU admission, or death in COVID-19 patients.

Current literature reports on strong predictors for the outcome *for example,* age is the strongest predictor for death, oxygen saturation is the strongest predictor for intubation.^35^ When these factors are added as inputs into the predictive models, the machine learning classifiers automatically choose these metrics instead of radiomics features. However, since the focus of this paper is to assess the predictive value of radiomic features for as markers for outcomes, we did not include non-imaging predictors within our prediction models. We considered ‘regressing out’ the influence from non-imaging predictors for each radiomic feature. However, the list of potential non-imaging predictors and its strength of association with each radiomic feature is variable. Also, arbitrary regressing out of too many non-imaging predictors (confounders) with weak associations will create artificial effects in the machine learning. Consequently, we adopted cross-validation as a safeguard against confounding effects. The confounding effect is always introduced by data sampling. When splitting data into learning and testing sample, the confounding relationship will change, *for example,* a confounder in learning sample will not likely be a confounder in testing sample. Therefore, if the model’s prediction accuracy can be repeated in the cross-validation process, it is indicative of minimal impact from confounding factors.

## Conclusion

This work represents the performance evaluation of CXR-based imaging metrics extracted from RT-PCR positive COVID-19 patients at admission to develop machine learning algorithms for predicting the need for ICU, the need for intubation, and mortality, respectively. Following validation using a larger cohort, early assessment of these predictors can help healthcare systems strategize resource management, particularly during a surge of cases. Future studies should explore rigorous validation strategies using deep learning methods within a multicenter setting.
