# Supplementary material for: Predicting clinical outcomes in COVID-19 using radiomics on chest radiographs
Source: Br J Radiol. 2022 Jul 8;94(1126):20210221. doi: 10.1259/bjr.20210221 (PMC9328073; doi:10.1259/bjr.20210221)
Supplement: Supplementary Material 1. [file bjr.20210221.suppl-01.docx]

**Supplement**

**Supplement Table A**: Sensitivity, specificity, positive predictive value, and negative predictive value for the 3 machine learning models across the three outcomes

| **ML Classifier** | **Sensitivity (Recall)** | **Specificity** | **PPV (Precision)** | **NPV** | **Prediction** |
| --- | --- | --- | --- | --- | --- |
| Random Forest | 0.64 95% CI (0.45, 0.83) | 0.65 95% CI (0.58, 0.73) | 0.25 95% CI (0.14, 0.35) | 0.91 95% CI (0.86, 0.97) | Mortality |
| Ada Boost | 0.68 95% CI (0.5, 0.86) | 0.69 95% CI (0.61, 0.77) | 0.28 95% CI (0.17, 0.39) | 0.92 95% CI (0.87, 0.97) | Mortality |
| ElasticNet | 0.56 95% CI (0.37, 0.75) | 0.58 95% CI (0.5, 0.67) | 0.19 95% CI (0.1, 0.28) | 0.88 95% CI (0.82, 0.95) | Mortality |
| Random Forest | 0.57 95% CI (0.46, 0.69) | 0.59 95% CI (0.49, 0.68) | 0.49 95% CI (0.38, 0.6) | 0.67 95% CI (0.57, 0.77) | Need for ICU |
| Ada Boost | 0.53 95% CI (0.41, 0.65) | 0.54 95% CI (0.44, 0.63) | 0.44 95% CI (0.33, 0.55) | 0.62 95% CI (0.52, 0.73) | Need for ICU |
| ElasticNet | 0.6 95% CI (0.49, 0.72) | 0.62 95% CI (0.52, 0.71) | 0.52 95% CI (0.41, 0.63) | 0.69 95% CI (0.6, 0.79) | Need for ICU |
| Random Forest | 0.64 95% CI (0.5, 0.78) | 0.65 95% CI (0.56, 0.73) | 0.4 95% CI (0.29, 0.52) | 0.83 95% CI (0.76, 0.91) | Need for intubation |
| Ada Boost | 0.67 95% CI (0.53, 0.8) | 0.66 95% CI (0.58, 0.75) | 0.42 95% CI (0.31, 0.54) | 0.84 95% CI (0.77, 0.92) | Need for intubation |
| ElasticNet | 0.62 95% CI (0.48, 0.76) | 0.63 95% CI (0.55, 0.72) | 0.38 95% CI (0.27, 0.5) | 0.82 95% CI (0.74, 0.9) | Need for intubation |

**Supplement Table B**: Description of patient cohort. Here, KMC: Keck Medical Center of USC; VHH: Verdugo Hills Hospital, and LAC USC: Los Angeles County + USC Medical Center.

|  | **Overall** | **LAC USC** | **KMC** | **VHH** | **P Value** |
| --- | --- | --- | --- | --- | --- |
| **No. of patients (n)** | 167 | 125 | 18 | 24 |  |
| **Age** | 55±17, 55  (43 to 68) | 52±15, 52  (43 to 62) | 62±18, 67  (50 to 75) | 67±21, 71  (62 to 82) | <0.01 |
| **Sex** |  |  |  |  |  |
| Male | 107 (64.07%) | 83 (66.4%) | 11 (61.11%) | 13 (54.17%) | 0.76 |
| Female | 59 (35.33%) | 41 (32.8%) | 7 (38.89%) | 11 (45.83%) |  |
| Other | 1 (0.6%) | 1 (0.8%) | 0 (0%) | 0 (0%) |  |
| **Ethnicity** |  |  |  |  |  |
| Hispanic Latino | 111 (66.47%) | 97 (77.6%) | 6 (33.33%) | 8 (33.33%) | <0.01 |
| Non-Latino | 40 (23.95%) | 15 (12%) | 10 (55.56%) | 15 (62.5%) |  |
| Unknown | 16 (9.58%) | 13 (10.4%) | 2 (11.11%) | 1 (4.17%) |  |
| **Mortality** |  |  |  |  |  |
| Survived | 142 (85.03%) | 110 (88%) | 16 (88.89%) | 16 (66.67%) | 0.02† |
| Deceased | 25 (14.97%) | 15 (12%) | 2 (11.11%) | 8 (33.33%) |  |
| **ICU Admission** |  |  |  |  |  |
| No ICU | 99 (59.28%) | 72 (57.6%) | 12 (66.67%) | 15 (62.5%) | 0.72 |
| ICU | 68 (40.72%) | 53 (42.4%) | 6 (33.33%) | 9 (37.5%) |  |
| **Intubation** |  |  |  |  |  |
| No intubation | 122 (73.05%) | 92 (73.6%) | 13 (72.22%) | 17 (70.83%) | 0.96 |
| Intubation | 45 (26.95%) | 33 (26.4%) | 5 (27.78%) | 7 (29.17%) |  |

**Supplement Table C:** Performance comparison of full vs. robust model

| **Model type** | **ML Classifier** | **StdErr** | **AUC** | **Prediction** |
| --- | --- | --- | --- | --- |
| Full Model | Random Forest | 0.044895089 | 0.56 95% CI: (0.49, 0.66) | Need for ICU |
| Full Model | Ada Boost | 0.045120518 | 0.61 95% CI: (0.49, 0.67) | Need for ICU |
| Full Model | ElasticNet | 0.043923378 | 0.61 95% CI: (0.53, 0.7) | Need for ICU |
| Full Model | Random Forest | 0.044154586 | 0.70 95% CI: (0.64, 0.81) | Need for intubation |
| Full Model | Ada Boost | 0.046021457 | 0.72 95% CI: (0.63, 0.81) | Need for intubation |
| Full Model | ElasticNet | 0.045796413 | 0.65 95% CI: (0.56, 0.74) | Need for intubation |
| Full Model | Random Forest | 0.058933064 | 0.69 95% CI: (0.59, 0.82) | Mortality |
| Full Model | Ada Boost | 0.065690227 | 0.71 95% CI: (0.58, 0.84) | Mortality |
| Full Model | ElasticNet | 0.05714838 | 0.61 95% CI: (0.5, 0.73) | Mortality |
| Robust Model | Random Forest | 0.043994116 | 0.63 95% CI: (0.54, 0.72) | Need for ICU |
| Robust Model | Ada Boost | 0.043290051 | 0.64 95% CI: (0.55, 0.72) | Need for ICU |
| Robust Model | ElasticNet | 0.043455528 | 0.62 95% CI: (0.54, 0.71) | Need for ICU |
| Robust Model | Random Forest | 0.036788775 | 0.8 95% CI: (0.73, 0.88) | Need for intubation |
| Robust Model | Ada Boost | 0.03976354 | 0.78 95% CI: (0.7, 0.86) | Need for intubation |
| Robust Model | ElasticNet | 0.047848094 | 0.64 95% CI: (0.55, 0.73) | Need for intubation |
| Robust Model | Random Forest | 0.058596127 | 0.76 95% CI: (0.64, 0.87) | Mortality |
| Robust Model | Ada Boost | 0.058663082 | 0.7 95% CI: (0.58, 0.81) | Mortality |
| Robust Model | ElasticNet | 0.053549411 | 0.67 95% CI: (0.57, 0.78) | Mortality |
